# Assessing the structural performance of a reinforced concrete beam: The effects of simulated rotation angle utilizing robot structural analysis software

**DOI:** 10.1016/j.mex.2024.102836

**Published:** 2024-07-03

**Authors:** Mustapha Kajja, Naima Taifi, Abdessamad Malaoui, Hassan Bita

**Affiliations:** a Polydisciplinary Faculty, Research in Physics and Engineering Sciences Laboratory, Sultan Moulay Slimane University, Beni Mellal 23000, Morocco; b Metrology and Information Processing Laboratory, Ibn Zohr University, Laayoune Technology School, Agadir 70000, Morocco

**Keywords:** Beam, Maximum bending moment, Maximum shear force, Maximum Deflection, finite element method investigated by Robot Structural Analysis Software

## Abstract

This study investigates the effect of the rotation angle in relation to the horizontal plane on the structural behavior of a reinforced concrete beam. Simulations were performed utilizing Autodesk's Robot Structural Analysis software for four different angles: 0°, 5°, 10°, and 15° The main objective is to evaluate the impact of these angles on bending moment, shear force, and deflection. The findings show that as the angle increases, the bending moments and shear forces decrease while the deflections increase.

• The methodology is a simulation study based on a finite element method.
• Autodesk's Robot Structural Analysis Software is the main tool of this study.

## Graphical abstract

**Figure:**
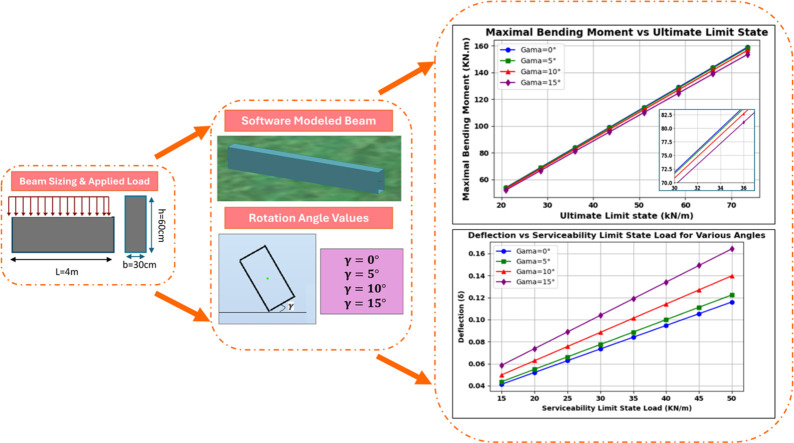

Specifications table

**Table:** 

Subject area:	Civil and Structural Engineering
More specific subject area:	Structural Behavior of a Reinforced Concrete Beam
Name of your method:	finite element method investigated by Robot Structural Analysis Software
Name and reference of original method:	Autodesk's Robot Structural Analysis Software
Resource availability:	N/A

## Background

In the fields of construction and civil engineering, structural elements are subjected to main loads, whether static or dynamic during their service life. The mechanical properties of these elements are modified by the effects of these loads [1] or by the addition of fibers [2]. The beam is an essential horizontal structural element for connecting one element to another [3]. It is used in the construction of buildings, bridges, and industrial structures. It is also distinguished by its shape and the materials used in its manufacturing. Its role is to back up vertical loads, transfer loads to supports, distribute loads efficiently and maintain the horizontal stability of structures [4]. The combination of concrete and steel in a reinforced concrete beam is effective in construction. Moreover, concrete is one of the most utilized materials for building [5] known for its strong resistance to compression loads, but it has low tensile strength whereas steel has high tensile strength, and this combination is an indispensable and widely used material in construction and civil engineering [6]. Maximum deflection is an essential parameter for analyzing the safety of structural elements and their service life [7], as it depends on several factors including the types and values of loads applied, beam geometry and materials, elasticity modulus, moment of inertia [8] as well as the type of boundary conditions. Several studies have been conducted about angles to describe the behavior of structural elements such as the angle of inclination of the tower of Pisa [9], the angle of shear, the angle of slope, and the rotation angle. Reinforced concrete beams can be subjected to loads from earthquakes, explosions, and ground movements during their service life, which can cause them to rotate in relation to the horizontal level. In such cases, it is significant to know the extent to which this rotation angle affects the behavior of reinforced concrete beams. It is an important parameter to consider when designing and building structures, as it helps to optimize the orientation of structural elements, enhance the building's rigidity and inertia, and improve its resistance to loads and strains. It is frequently used in civil engineering to guarantee structural stability and safety.

There are several methods, both analytical and numerical for modeling concrete structures [10]. To obtain approximate solutions, the finite element method simulates structural elements with boundary conditions [11]. It is the most widely used method in recent years for solving practical engineering problems [12], the analysis of structural systems [13] and commonly utilized for simulating reinforced concrete elements by Robot Structural Analysis software, which is used to analyze, dimension and model complex structures such as bridges, towers, factories, reservoirs, swimming pools and stadiums [14]. It is a simulation tool used by civil engineers to analyze structural elements and mechanics [15]. Time-saving and easy-to-calculate modules are also included, offering static, dynamic, and modal analysis as well as linear and non-linear material analysis [16]. The use of the software allows to produce project documentation [17].

This study attempts to simulate the behavior of a rectangular reinforced concrete beam subjected to uniformly distributed loading using Robot Structural Analysis software. The parameter used in this modeling is the rotation angle of the beam with respect to the horizontal level. The results show that the angle of rotation has significant effects on maximum bending moment, maximum shear force and maximum deflection.

## Method details

### Pre-Dimensioning of the beam

**Fig. 1:**
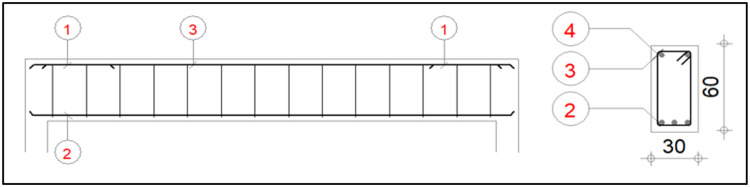
Beam Reinforcement Plan.

**Fig. 2:**
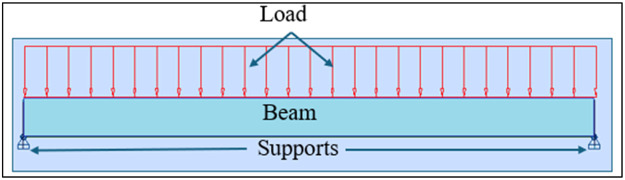
A Concrete Reinforcement Beam Model.

The reinforced concrete beam studied is one that has the following characteristics: length is 4 m, width is 30 cm and height is 60 cm on two rotulas supports of which the width is 0,2 m. The beam is reinforced with high strength steel bars of grade 400. The geometric characteristics and reinforcement plan of the beam are carried out in accordance with Eurocode 2 and are presented in Fig. 1. For the bars with diameters of 8, 12 and 14 mm are used for longitudinal high strength reinforcement at the top and bottom of the beam; for 6 mm diameter bars are used as transversal high strength reinforcement. Table 1 provides all the necessary details about these bars. Fig. 2 shows the model of reinforced concrete beam studied in Robot Structural Analysis.

**Table 1:** Beam Reinforcement Details.

Position	Reinforcement Type	Steel Grade	Number	Diameter
1	Main-top	High strength 400	6	Ø12
2	Main bottom	High strength 400	3	Ø12
3	assembly-top	High strength 400	2	Ø8
4	transverse-main	High strength 400	14	Ø8

### Material properties

Table 2 shows the mechanical properties of concrete and steel for the beam model. The cylindrical compressive strength of concrete is f_cm_=25 MPa. This value is usually utilized in studies because it is the most readily available concrete in the market as it conforms with Moroccan standard MN.10.1.OO8 [18].The modulus of elasticity of concrete is calculated by relationship 1 [20] and the shear modulus is calculated by relationship 2 [19].

(1) $$E=22000\cdot {\left(f_{cm}/10\right)}^{0.3}$$

(2) $$G=\frac{E}{2\left(1+\vartheta \right)}\;$$

**Table 2:** Input Parameters for Concrete and Steel.

Properties	Materials			
	Concrete		Steel	
	Value	Unit	Value	Unit
Compressive Strength (fcm)	25	MPa	–	–
Young Modulus (E)	28,960,4	MPa	21	GPa
poisson Ratio ((ϑ)	0,2	–	0,3	–
Shear Modulus (G)	12,066,8	MPa	80,800	MPa

### Beam loading

**Fig. 3:**
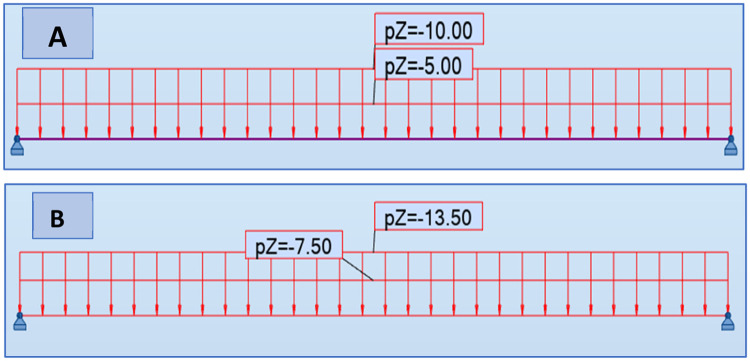
Robot Structural Analysis Model of the Beam in the Case of *Q* = 5KN/m and γ=0°

Fig. 2 above shows the beam model, the reaction supports, and the applied load that are uniformly distributed to study the structural behavior of the beam. The self-weight of the beam is equal to 4.5 kN, the permanent load (*G* = 10 kN/m) and the live load (Q) which is applied to the beam vary from 5 kN/m to 40 kN/m. The combination of loads at the ultimate limit state is determined by the following relationship: 1.35G+1.5Q and the combination for the serviceability limit state in which the relationship used is *G* + *Q* [19]. The results that are noted for each 5 kN/m increase in live load. For table 3, it shows the different loads applied; also, Fig. 3 shows the Robot Structural Analysis model of the beam for the case of 5 kN/m live load and γ=0°

**Table 3:** Dead Loads, Live Loads, and Combined Loads at Limit States.

SW (kN)	G(kN/m)	Q(kN/m)	Ultimate Limit State Load (kN/m)	Serviceability Limit State Load (kN/m)
4,5	10	5	21	15
4,5	10	10	28,5	20
4,5	10	15	36	25
4,5	10	20	43,5	30
4,5	10	25	51	35
4,5	10	30	58,5	40
4,5	10	35	66	45
4,5	10	40	73,5	50

## Method validation

Overall, it is noted that the rotation angle has a significant impact on the bending moment, the shear force and the deflection. More details are to be discussed in the following sections:

### Effect of rotation angle on maximum bending moment

**Fig. 4:**
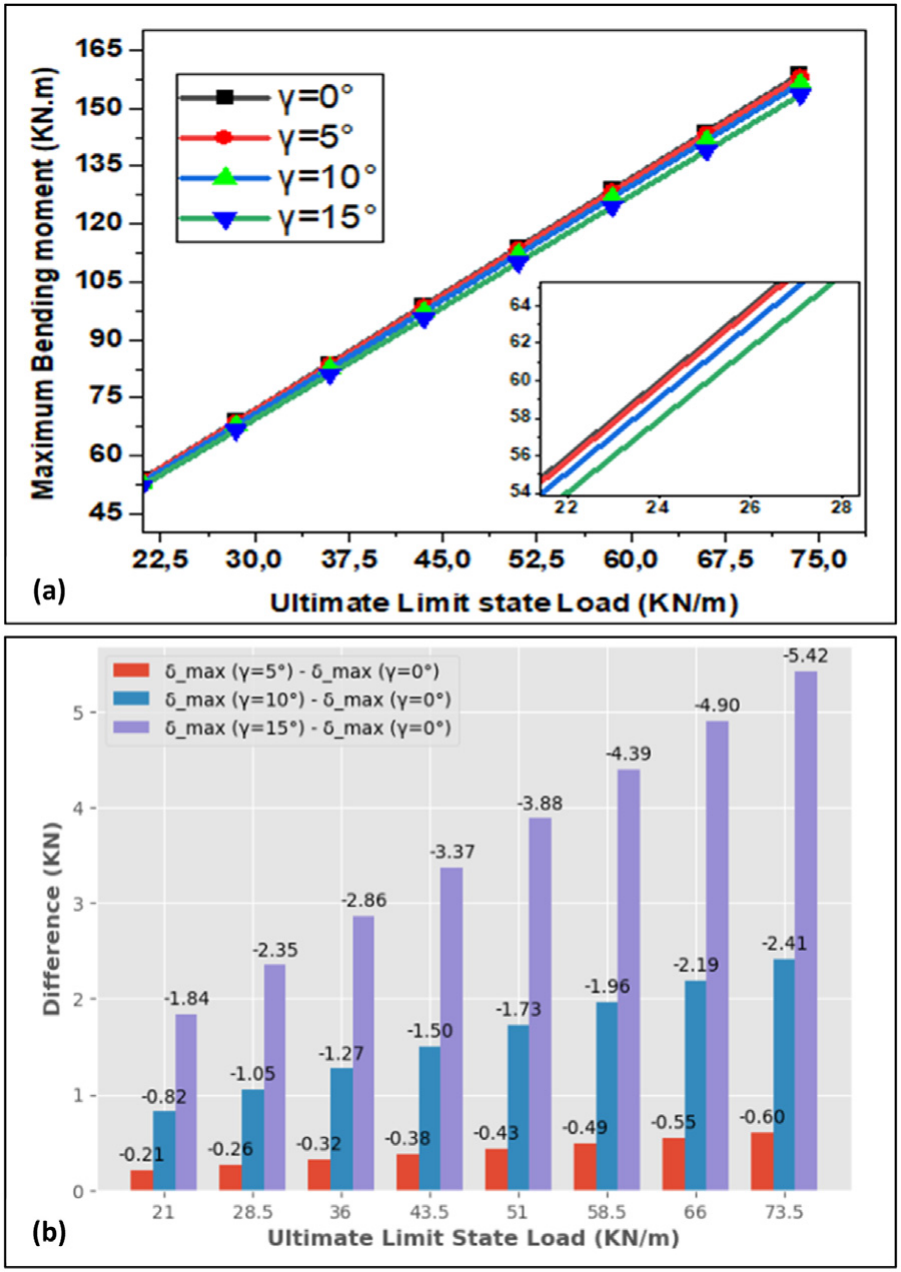
(a) Bending Moment for Ultimate Limit State and (b) Observed Difference of Rotation Angle.

**Fig. 5:**
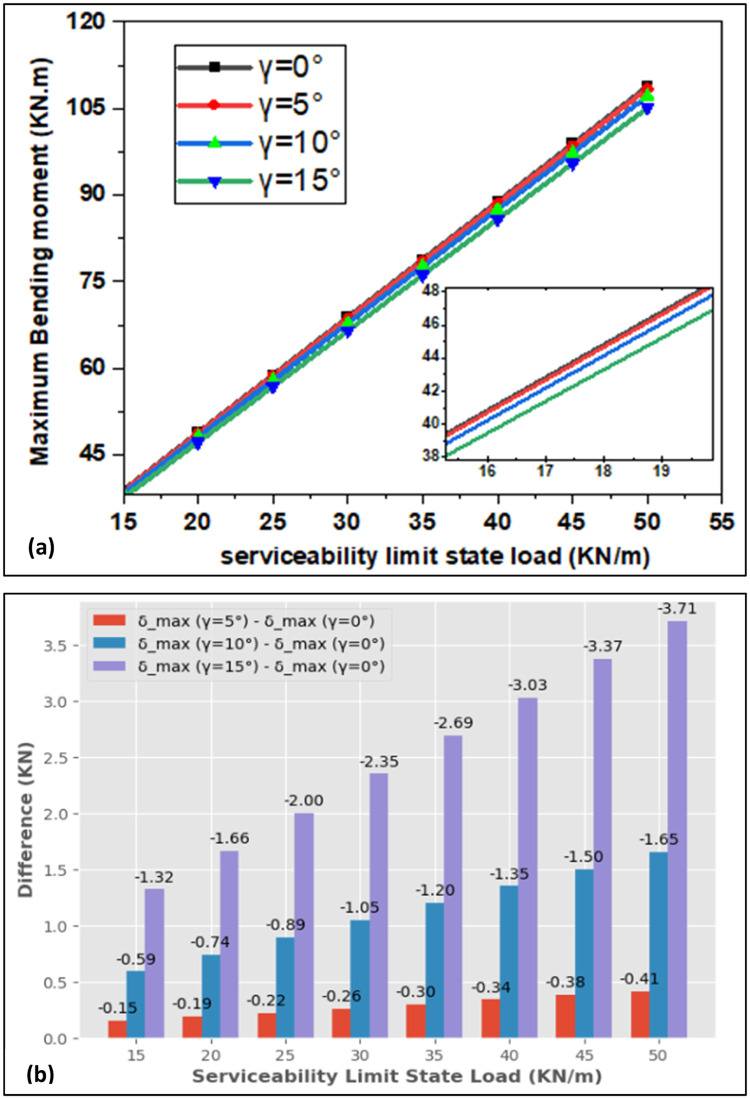
(a) Bending Moment for Serviceability Limit State and (b) Observed Difference of Rotation Angle.

**Fig. 6:**
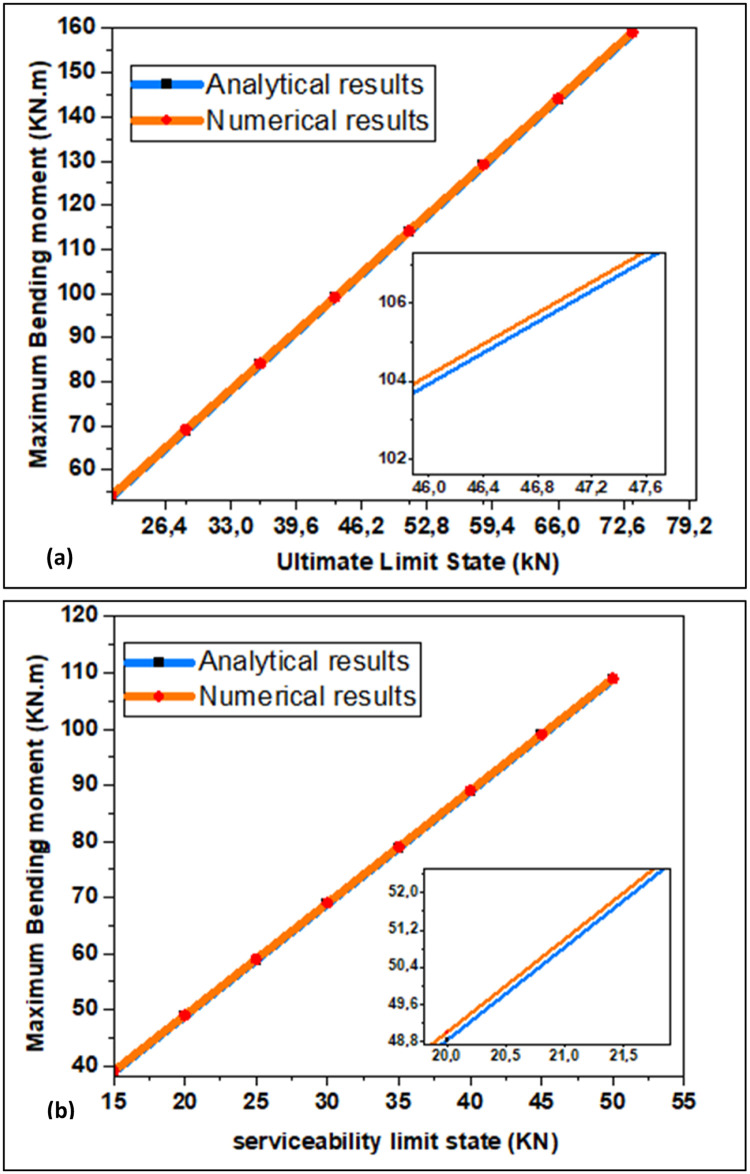
Comparison Between Analytical and Numerical Results for γ=0° in the case (a) Ultimate limit state (b) serviceability limit state.

Fig. 4 portrays the maximum bending moment (Mmax) as a function of the ultimate limit state load with observed difference; also, Fig. 5 conveys the maximum bending moment as a function of serviceability limit state load with observed difference after increasing the live load by 5KN/m steps. When the ultimate limit state load is elevated, the bending moment grows linearly. When the maximum bending moment calculated by the relation $M_{\text{fmax}}=\frac{P.L^{2}}{8}$ [21,22] is compared with the result found by the simulation for γ=0°, a difference of 0.23 kN.m is observed for the ultimate limit state case whereas 0.17 kN.m is observed for the serviceability limit state case. Fig. 6 shows the comparison between analytical and numerical results; as the rotation angle rises, the maximum bending moment decreases as function of load. The same results are obtained for the serviceability limit load case. As the load rises, the maximum bending moment goes up, and as the rotation angle increases, maximum bending moment goes down. When the design is optimized, the risk of beam failure is reduced; so, this incorporates minimizing the bending moment within the beam. In brief, there is a causal and coherent relationship between decreasing maximum bending moment with increasing rotation angle and the design to minimize the risk of beam failure and improve the service life of the beam.

### Effect of angle rotation on maximum shear force

**Fig. 7:**
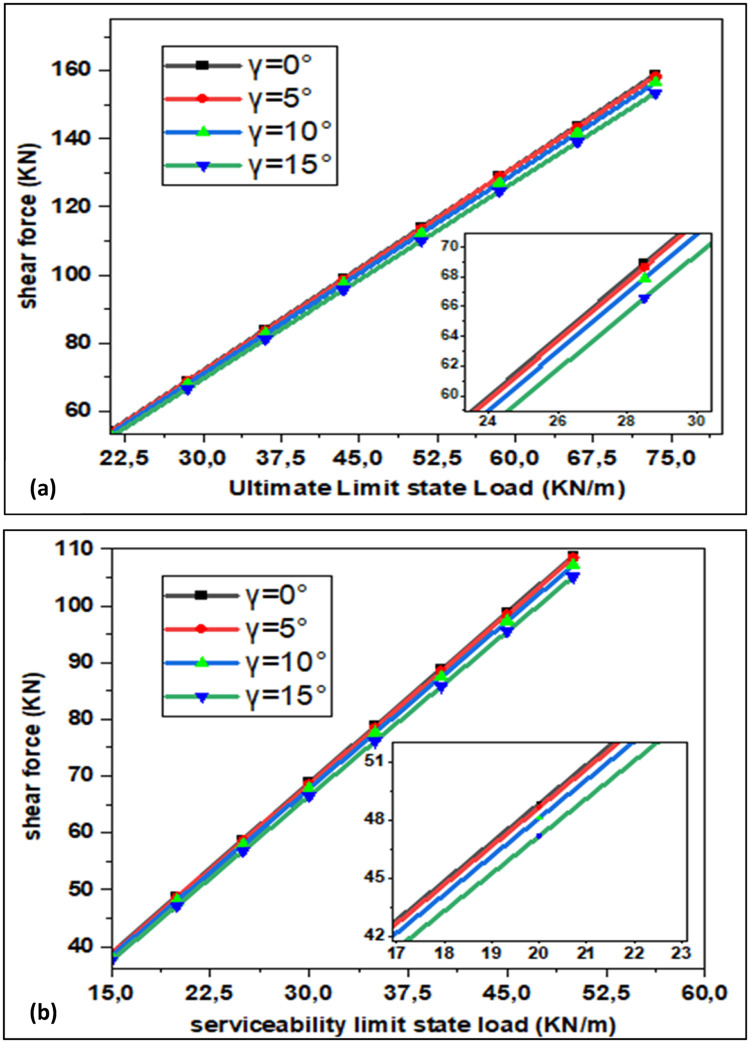
Shear Force in the Case of (a) Ultimate Limit State and (b) Serviceability Limit State.

The graphical representation of maximum shear force values as function of loads in the ultimate limit state and in the service limit state is shown in Fig. 7. The relationship between shear force and loads is linear, which means that the shear force increases proportionally along with the increase in loads. The comparison of the results found numerically for γ =0° with those found by the relationship $V_{max}=\frac{P.L}{2}$ [16], shows that the difference for the maximum shear force is the same as the maximum bending moment. Hence, as the rotation angle goes up, maximum shear force decreases since M_max_ is equal to Vmax in this case.

### Effect of rotation angle on maximum deflection

**Fig. 8:**
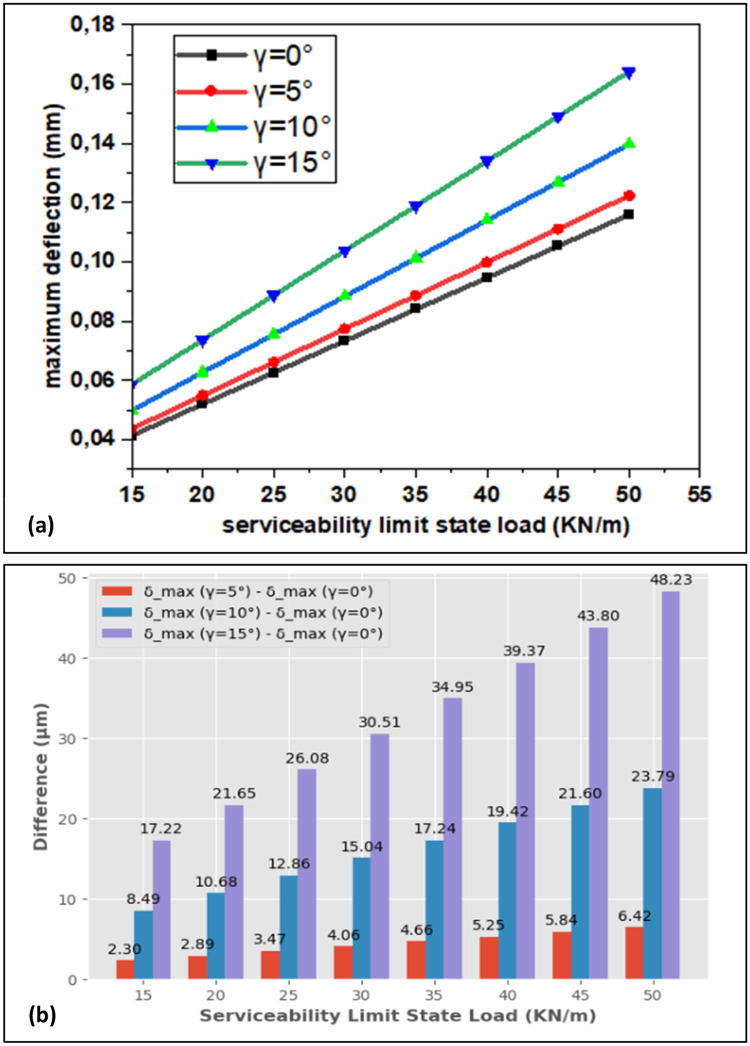
(a) Maximum Deflection for Serviceability Limit State Case and (b) Observed Difference for Rotation Angle.

The relationship between load and deflection for rotation angle with the values of γ=0°, γ= 5°, γ= 10° and γ= 15° is shown in Fig. 8 for serviceability limit state. The maximum deflection rises with the increasing loads and grows up with the increasing of rotation angle from γ=0° to γ=15° The maximum deflection makes an increase from17.22 μm to 48. 23μm for the serviceability limit state. As a result, the rotation angle impacts the maximum deflection of the beam and does not exceed the criteria of Eurocode 2 standards for 5°, 10° and 15° angle rotation.

## Limitations

‘None’.

## Ethics statements

The ethical guidelines comply with by the authors. No data was collected from social media platforms.

## CRediT author statement

The data used to support the findings of this study are available from the corresponding author upon request. CONTRIBUTIONS All authors contributed to the study conception, design, data collection, analyses, manuscript writing, and/or revisions. All authors read and approved the final manuscript.

## Declaration of competing interest

The authors declare that they have no known competing financial interests or personal relationships that could have appeared to influence the work reported in this paper.

## Data Availability

the data used are based an investigation method by means of Robot structural Analysis Software.
